# Salivary Microbiome in Adenoid Cystic Carcinoma Detected by 16S rRNA Sequencing and Shotgun Metagenomics

**DOI:** 10.3389/fcimb.2021.774453

**Published:** 2021-12-14

**Authors:** Qian Jiang, Xing Liu, Qifen Yang, Liang Chen, Deqin Yang

**Affiliations:** ^1^ Stomatological Hospital of Chongqing Medical University, Chongqing, China; ^2^ Chongqing Key Laboratory of Oral Diseases and Biomedical Sciences, Chongqing, China; ^3^ Chongqing Municipal Key Laboratory of Oral Biomedical Engineering of Higher Education, Chongqing, China

**Keywords:** microbiota, salivary adenoid cystic carcinoma, oral cancer, 16S rRNA sequencing, metagenomics, bioinformatics

## Abstract

Microorganisms are confirmed to be closely related to the occurrence and development of cancers in human beings. However, there has been no published report detailing relationships between the oral microbiota and salivary adenoid cystic carcinoma (SACC). In this study, unstimulated saliva was collected from 13 SACC patients and 10 healthy controls. The microbial diversities, compositions and functions were comprehensively analyzed after 16S rRNA sequencing and whole-genome shotgun metagenomic sequencing. The alpha diversity showed no significant difference between SACC patients and healthy controls, while beta diversity showed a separation trend. The SACC patients showed higher abundances of *Streptococcus* and *Rothia*, while *Prevotella* and *Alloprevotella* were more abundant in healthy controls. The prevalent KEGG pathways, carbohydrate-active enzymes, antibiotic resistances and virulence factors as well as the biomarkers in SACC were determined by functional gene analysis. Our study preliminarily investigated the salivary microbiome of SACC patients compared with healthy controls and might be the basis for further studies on novel diagnostic and treatment strategies.

## Introduction

Adenoid cystic carcinoma (ACC) is a rare tumor typically arising from salivary glands, accounting for about 1% of all head and neck malignancies and about 10% of all tumors of the salivary glands (Dodd and Slevin, 2006; Coca-Pelaz et al., 2015). It is characterized by unpredictable growth, extensive perineural invasion, high risk of metastasis, and low long-term survival rate (Adelstein et al., 2012; Fu et al., 2019). Standard treatment, including surgery plus intensive radiation treatment, often requires extensive reconstructive surgery and results in poor long-term prognosis and significant morbidity. Chemotherapy is generally reserved for the palliative treatment of symptomatic locally recurrent or metastatic disease that is not amenable to further surgery or radiation, but effective chemotherapy regimen is limited and unclear (Laurie et al., 2011).

There is growing evidence showing the critical role played by microorganisms in carcinogenesis. Microbes induce an estimated 20% of all the fatal cancers in human beings and a large number of bacterial species are associate with cancers (de Martel et al., 2012). The role of gut microbiota, especially *Helicobacter pylori*, in promoting gastric cancer and colorectal cancer has been well elucidated (Marshall and Windsor, 2005; Wassenaar, 2018). Some other findings of related researches are *Salmonella typhi* and gall bladder cancer (Shukla et al., 2000), *Veillonella* and *Megasphaera* and lung cancer (Lee et al., 2016), *Sphingomonas* and *Methylbacterium* and breast cancer (Xuan et al., 2014), *Pseudomonas* and *Elizabethkingia* and pancreatic cancer (Pushalkar et al., 2018), *Bartonella* species and tumor vessel generation (Dehio, 2005).

Oral cavity, as one of the most diverse microbial habitats in the human body, harbors more than 700 bacterial species. Along with the progress in high-throughput sequencing technique, previous studies have shown significant changes in the abundance of oral microbiota in oral cancer patients compared to healthy individuals (Lim et al., 2017; Hayes et al., 2018). Saliva microbiome was suggested to reflect health and disease status under certain circumstances, making it a promising biomonitor for disease diagnostics and prognostic (Guerrero-Preston et al., 2016; Bel'skaya et al., 2020). Several studies have investigated the microbiota of cancer tissue and saliva in oral squamous cell carcinoma (OSCC), revealed its relationships to the sites and stages of cancer, and indicated that oral microbes were potential indicators for the initiation and development of OSCC (Yang et al., 2018; Healy and Moran, 2019; Zhang et al., 2019). However, there is no comprehensive study on the relationship of salivary adenoid cystic carcinoma (SACC) and oral microbiome.

## Materials And Methods

### Subjects and Sampling

Subjects were recruited from the Affiliated Hospital of Stomatology and the First Affiliated Hospital of Chongqing Medical University in Chongqing, China. Patients diagnosed with salivary adenoid cystic carcinoma *via* pathology without chemotherapy, radiotherapy or surgery were enrolled into the SACC group, and healthy control individuals were matched for age and gender to them. Subjects were excluded if they (i) had other bacterial infectious oral disease, (ii) had removable partial denture, bridge or implant, (iii) had systemic disease, (iv) were pregnant or lactating women, (v) had received systemic antibiotics within 3 months, or (vi) had received local antibiotic treatment within 2 weeks.

Thirteen SACC patients (group S) and 10 healthy individuals (group H) were enrolled in our study, as presented in **Table 1**. Saliva was collected from each subject before breakfast and tooth brushing in the morning. Subjects were required to rinse their mouth with sterile saline before sample collection to avoid the presence of contaminants. For each participant, 5 mL of non-stimulated saliva was collected into a sterile test tube on ice, and immediately stored at -80°C until further processing.

**Table 1:** The demographic data of subjects.

Variable	Group S	Group H	P value
**Age (years)**	57.00 ± 1.53	57.60 ± 1.81	0.802
**Gender**			>0.999
Male	7	5	
Female	6	5	
**Tumor site**			
Parotid	6		
Submandibular	2		
Sublingual	2		
Palate	3		
**Tumor stage**			
T2 N0 M0	5		>0.999
T3 N0 M0	6		
T4 N0 M0	2		
**Tobacco**			
Yes	7	5	
No	6	5	
**Alcohol**			0.685
Yes	8	5	
No	5	5	

This study was approved by the Ethics committee of Chongqing Medical University (ethical approval number: CQHS-IRB-2017-01). Written informed consent was obtained from each participant.

### DNA Preparation, 16S rRNA Sequencing and Metagenomic Sequencing

Total DNA was extracted with the FastDNA^®^ Spin Kit for Soil (MP Biomedicals, Solon, USA) according to the manufacturer’s protocol. DNA concentration was assessed by Nanodrop 2000 (Thermo Scientific, Wilmington, USA) and DNA quality was determined by 1% agarose gel electrophoresis.

For 16S rRNA sequencing, primers 338F (5’-ACTCCTACGGGAGGCAGCAG-3’) and 806R (5’-GGACTACHVGGGTWTCTAAT-3’) were used to amplify the V3-V4 hypervariable regions of the bacterial 16S rRNA gene using PCR on a thermocycler (GeneAmp 9700, ABI, USA). The final PCR products were extracted from a 2% agarose gel, purified by the AxyPrep DNA Gel Extraction Kit (Axygen Biosciences, Union City, CA, USA) and quantified using QuantiFluorTM -ST (Promega, USA). When purification and quantification were finished, a compound of amplicons were merged into equimolar concentrations and paired-end sequenced (2 × 300) on Illumina MiSeq PE300 (Illumina, San Diego, USA).

For metagenomic shotgun sequencing, total DNA prepared from saliva samples were sheared on Covaris S220 (Covaris, Woburn, MA, USA) to an average size of 400 bp. Library preparation was performed according to Illumina’s TruSeq Nano DNA Sample Preparation protocol, followed by sequencing on Illumina HiSeq 2500 (Illumina, San Diego, USA).

The raw reads were deposited into the NCBI Sequence Read Archive (SRA) database (Accession Number: SRP336507).

### Bioinformatics Analysis

After 16S rRNA sequencing, operational taxonomic units (OTUs) were clustered with 97% similarity cutoff using UPARSE (version 7.1 http://drive5.com/uparse/). By comparing the RDP Classifier algorithm (http://rdp.cme.msu.edu/) against the Silva (SSU123) 16S rRNA database, the taxonomy of each 16S rRNA gene sequence was analyzed based on a 70% confidence threshold. Bioinformatics analyses were conducted using QIIME (version 1.9.1). Alpha diversity analysis was performed by calculating indices of Shannon, Simpson, Chao and ACE using Mothur (version 1.31.2). Differences between SACC patients and healthy controls were examined by Student’s *t* test. Beta diversity analysis was performed by principal coordinates analysis (PCoA) based on Bray-Curtis distances at the OTU level. Difference between the two groups was tested using PERMANOVA statistical test.

To characterize the taxonomic composition of metagenomic dataset, sequences were aligned against the NCBI microbial NR database (ftp.ncbi.nlm.nih.gov/blast/db/) using DIAMOND software (version 0.8.35). The functional annotation was assigned to the unigenes by blasting against the KEGG orthology database. The databases of CAZy (version 6, http://www.cazy.org/), CARD (version 1.1.3, https://card.mcmaster.ca/), and VFDB (version 2016.03, http://www.mgc.ac.cn/VFs/main.htm) were also used to analyze the specific functions of salivary microbiome, including carbohydrate-active enzymes (CAZymes), antibiotic resistances, and virulence factors. The predominant members in the taxonomic and functional compositions of salivary microbiome with significant differences between SACC patients and healthy individuals were determined using Wilcoxon rank-sum tests. LDA Effect Size (LEfSe) was used to identify high-dimensional biomarkers.

Differences were considered significant when P < 0.05 and highly significant when P < 0.01. SPSS 25.0 software (SPSS Inc, Chicago, IL, USA) was used for statistical analysis.

## Results

### Sequences Information and Taxonomic Annotation

A total of 404,647 valid reads were obtained after 16S rRNA sequencing, with an average of 17,593 for each sample. The clustering of qualified sequences at 97% identity resulted in 16 phyla, 27 classes, 45 orders, 69 families, 136 genera and 259 species. The rarefaction curves (**Appendix Figure 1**) achieved the even stage, suggesting that the sampling was almost complete.

After metagenomic sequencing, a total of 2,130,133,012 clean reads were generated from 2,154,848,364 raw reads, and the mean number of reads for each sample was 92,614,478. After taxonomic annotation, bacteria, fungi, viruses, and archaea could be determined in salivary microbiome, with a total of 123 phyla, 237 classes, 516 orders, 938 families, 2539 genera and 11300 species detected.

### Alpha and Beta Diversity Analysis Based on 16S rRNA Sequencing

**Figure 1:**
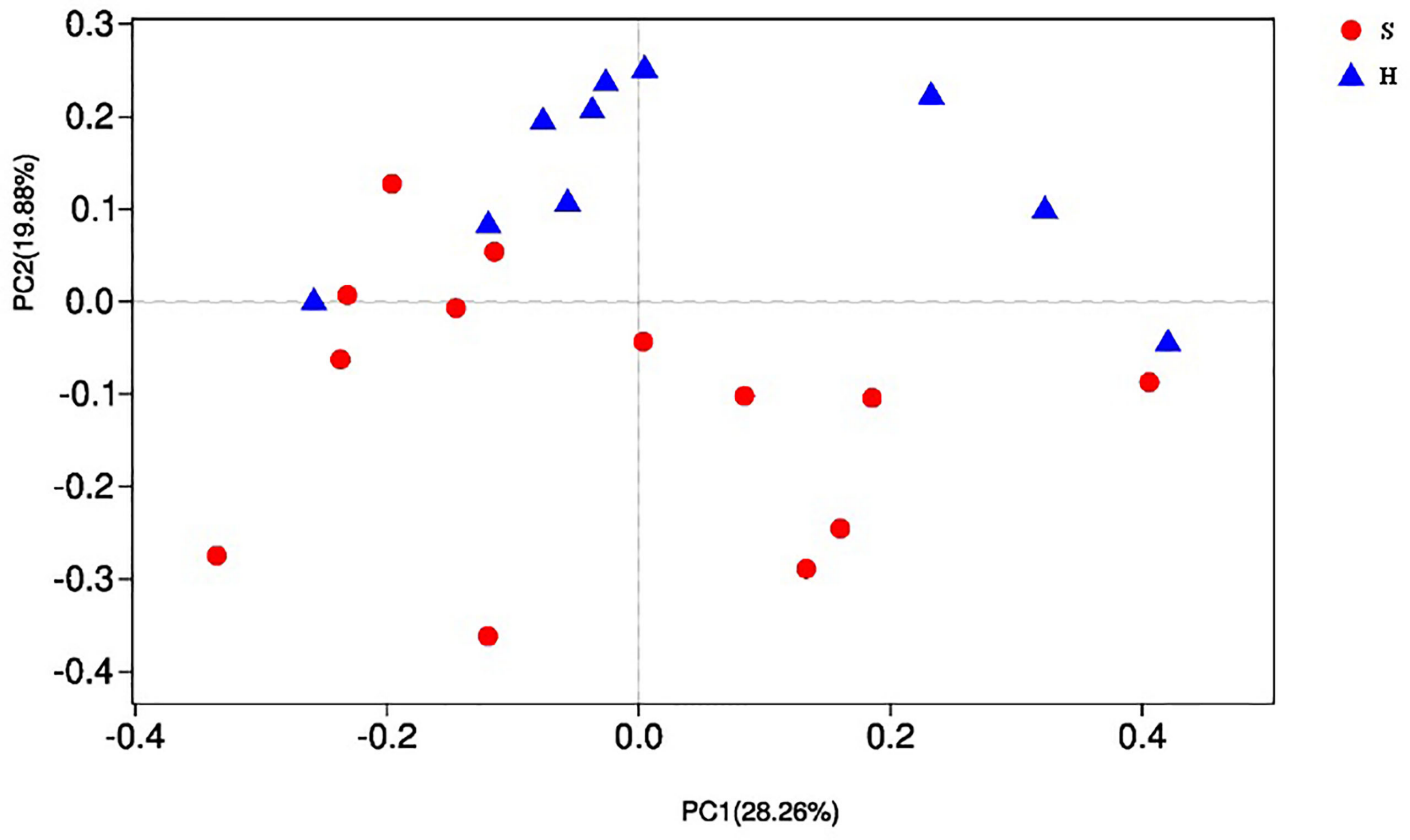
Beta diversity by PCoA. Each sample is represented by a dot, and different colors represent different groups. As for each sample, the first two main coordinates, namely PC1 and PC2 were depicted. PC1 explained 28.26% of the variation observed, and PC2 explained 19.88% of the variation.

As shown in **Table 2**, the alpha diversity indices of Shannon, Simpson, ACE and Chao were calculated and compared between the two groups. No significant difference was observed in bacterial diversity and richness (P>0.05). Beta diversity was analyzed by PCoA. A separation trend between the two groups could be observed (**Figure 1**), showing that the phylogenetic distance significantly separated SACC patients from healthy controls (P<0.05).

**Table 2:** Alpha diversity indices.

Group	Shannon	Simpson	ACE	Chao
**SACC patients** (*n* =13)	3.08 ± 0.44	0.13 ± 0.08	193.18 ± 27.52	191.41 ± 34.00
**Healthy controls** (*n* =10)	3.21 ± 0.39	0.09 ± 0.05	197.30 ± 38.76	199.59 ± 40.66
**P value**	0.49	0.27	0.76	0.60

Each applicable value is mean ± Sd. Richness estimators (Chao and ACE) and diversity estimators (Shannon and Simpson) were calculated. Differences between the two groups were examined by Student’s t test.

### Microbial Compositions Determined by Metagenomic Sequencing

**Figure 2:**
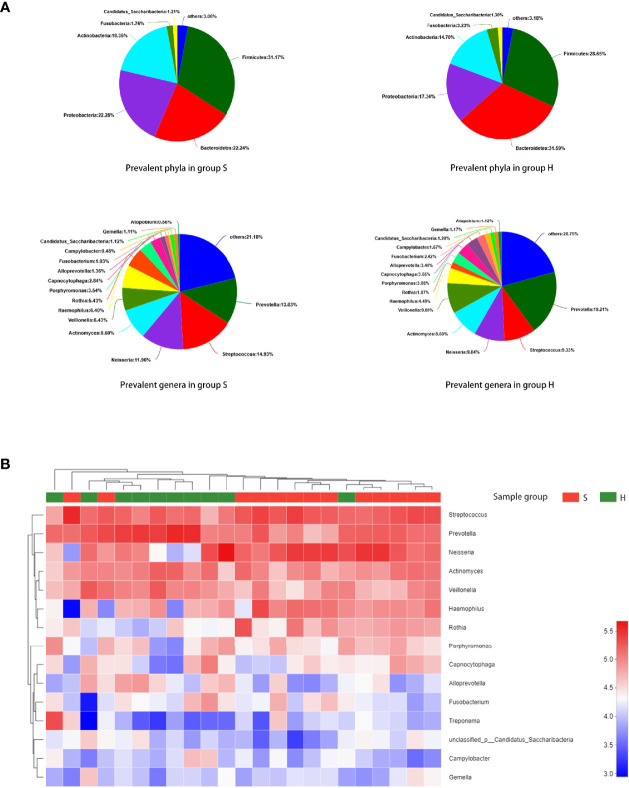
Taxonomic composition of the salivary microbiome. **(A)** The predominant taxa (relative abundance >1% on average) in each group are shown. **(B)** Heatmap analysis. Each column represents a sample and each row represents a genus. The cluster trees of genera and samples are shown on the left and upper sides respectively. Different colors represent different relative abundances.

**Figure 3:**
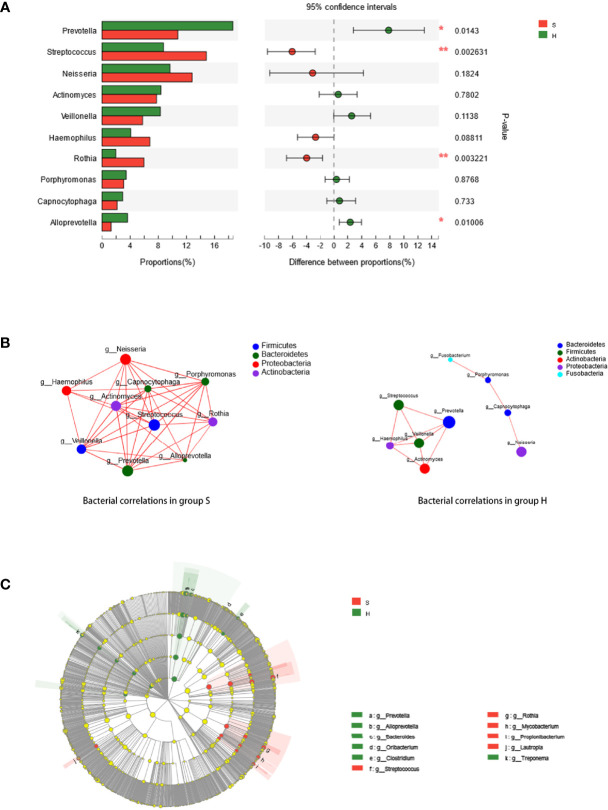
**(A)** Wilcoxon rank-sum test bar plot. Relative abundances of the ten most prevalent genera are compared between group S and (H) * represents a significant difference (P < 0.05) and ** represents a highly significant difference (P < 0.01). **(B)** Correlations of prevalent genera. Ten richest genera were shown by co-occurrence analysis. The size of the node is proportional to the genera abundance. Node color corresponds to phylum taxonomic classification. **(C)** Potential biomarkers defined by LEfSe. Cladogram for taxonomic representation of significant differences between group S and H were shown. The colored nodes from the inner to the outer circles represent taxa from the phylum to genus level. The significantly different bacteria are signified by different colors representing the two groups.

The predominant phyla and genera are shown in **Figure 2A**. The six most abundant phyla were *Firmicutes* (31.2% in group S and 28.6% in group H), *Bacteroidetes* (22.2% in group S and 31.6% in group H), *Proteobacteria* (22.3% in group S and 17.3% in group H), *Actinobacteria* (18.3% in group S and 14.7% in group H), *Fusobacteria* (1.8% in group S and 3.2% in group H), and *Candidatus Saccharibacteria* (also known as *Candidate division TM7*, 1.2% in group S and 1.3% in group H). At the genus level, *Prevotella, Streptococcus, Neisseria, Actinomyces, Veillonella, Haemophilus, Rothia, Porphyromonas, Capnocytophaga* and *Alloprevotella* were the most prevalent. **Figure 2B** represents a heatmap showing the relative abundances of these predominant genera in each sample, as well as the cluster trees of the genera and samples. The microbial compositions of SACC patients and healthy controls were mostly separated, consistent with the result of beta diversity based on 16S rRNA sequencing. As shown in **Figure 3A**, among these predominant genera, *Streptococcus* and *Rothia* showed higher relative abundances in SACC, while *Prevotella* and *Alloprevotella* were more abundant in healthy controls (Wilcoxon rank-sum tests, P<0.05). Co-occurrence analysis was performed to recognize interactions among these prevalent genera (**Figure 3B**). Compared to healthy individuals, the salivary microbiota of SACC patients exhibited more complex and aggregated relationships among each other.

The taxa that most likely explain the differences were defined by LEfSe. **Figure 3C** shows cladograms representing the potential biomarkers of different groups. *Streptococcus, Rothia, Lautropia, Mycobacterium* and *Propionibacterium* were remarkably enriched in SACC patients, while *Prevotella, Alloprevotella, Treponema, Bacteroides, Oribacterium* and *Clostridium* were significantly more abundant in healthy controls.

### Microbial Functions Determined by Metagenomic Sequencing

**Figure 4:**
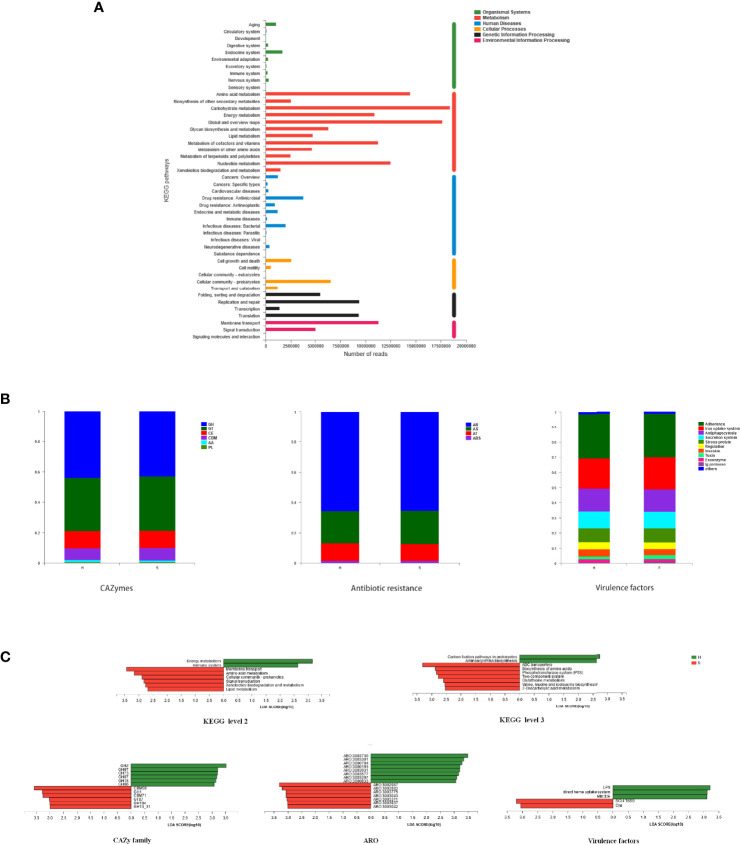
Functional genes of the salivary microbiome. **(A)** Genes related to KEGG pathways. Each branch represents a KEGG pathway on level 2, and different colors represent different KEGG level 1 functions. **(B)** Genes related to specific functions. The predominant taxa (relative abundance>2% on average) of CAZyme class genes, genes related to antibiotic resistance on class level, and genes related to virulence factors on level 2 are shown. **(C)** Potential biomarkers defined by LDA scores. Differentially abundant KEGG pathways on level 2 and level 3 (LDA > 2.5, P < 0.05) and differentially abundant CAZyme families, AROs, and virulence factors (LDA > 3, P < 0.05) were shown.

Microbial KEGG Orthologs (KO) were identified in salivary metagenomes. **Figure 4A** shows vigorous metabolism, dominated by carbohydrate metabolism, global and overview metabolism, amino acid metabolism, nucleotide metabolism, metabolism of cofactors and vitamins, energy metabolism, and glycan metabolism. Other predominant KEGG pathways are mainly associated to membrane transport, replication & repair, translation, cellular community, folding & sorting & degradation, and signal transduction.

The relative abundances of functional genes related to specific functions are shown in **Figure 4B**. As indicators of overall genetic potential for carbohydrate and lignin degradation, 431 CAZy gene families were examined. Glycoside hydrolases (GH) and glycosyl transferases (GT) enzymes were predominant, followed by carbohydrate esterases (CE) and carbohydrate-binding modules (CBM), with similar abundances in SACC patients and healthy controls. The most abundant families were GT2, GT4, GT51, CE1, GH2, CBM50, GH23 and GH20. Functional compositions related to antibiotic resistances were found to be similar in the two groups by aligning sequences against CARD database. The most abundant antibiotic resistance ontologies (AROs) were mfd, sav1866, rpoC, macB, alaS, PBP1a and rpoB. Virulence factors related to adherence, iron uptake system, antiphagocytosis, secretion system, regulation, invasion, toxin, exoenzyme and Ig protease were the most abundant.

**Figure 5:**
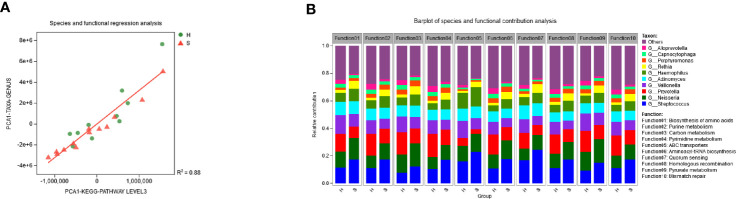
Relationships of microbiota and functions. **(A)** The microbial and functional regression analysis. The microbial community diversity was based on genus level and the functional diversity was based on KEGG pathway level 3. **(B)** Relative contribution of different genera to identified the enriched functional attributes in the two groups.

The regression analysis showed that the diversity of the functional profiles from the metagenomic data was significantly correlated with the diversity of microbial communities across the samples (R^2^ = 0.88, P < 0.01) (**Figure 5A**). The taxonomic origin of enriched functional attributes is visualized in **Figure 5B**. *Streptococcus, Neisseria*, and *Prevotella* were the main contributors of these functions. *Stretococcus* contributed significantly more to group S while *Prevotella* contributed more to group H (paired t test, P<0.05). *Neisseria* was the greatest contributor in carbon metabolism and pyruvate metabolism.

LEfSE was also performed to define the functional differences in the two groups. As shown in **Figure 4C**, the SACC group was associated with several KEGG pathways, including membrane transport, amino acid metabolism, cellular community, signal transduction, xenobiotics biodegradation & metabolism, and lipid metabolism. And the healthy group was more correlated with pathways such as energy metabolism and immune system. More specifically, ABC transporters, biosynthesis of amino acids, phosphotransferase system, two-component system, glutathione metabolism, biosynthesis of valine & leucine & isoleucine, and 2-Oxocarboxylic acid metabolism were remarkably enriched in SACC, while carbon fixation pathways and aminoacyl-tRNA biosynthesis were significantly enriched in healthy controls. Some CAZymes such as CBM50, GH1, CBM71, GT87, GH104 and GH13_31 were detected to be enriched in group S, while GH2, GH97, GH73, GH87, GH18 and GH66 were enriched in group H. Based on CARD database, bcrA, lmrD, murA, PBP2x, PBP2b and parC were significantly enriched in group S, while ileS, gyrB, cmeB, tetT, gyrA, PmrE and evgS were more abundant in group H. Analysis of virulence factors showed that SCI-I T6SS and Cya were enriched in SACC, while LPS, MtrCDE and direct heme uptake system were enriched in healthy controls. All above analysis may demonstrate the potential mechanisms of SACC.

## Discussion

The oral cavity is an intricate environment composed of multiple structures and tissues. While each structure performs a unique function, all are colonized by microbiota and immersed in salivary fluids. Previous studies have shown that salivary constituents may actually be effective indicators for disorders ranging from cancer to infectious diseases (Bonne and Wong, 2012; Yoshizawa et al., 2013). Using high-throughput sequencing technology to sequence 16S rRNA gene in saliva can quickly identify those microorganisms which have not been cultured, and distinguish the microbial community structure. However, despite its widely application, 16S rRNA sequencing has some limitations. Taxa are assigned based on the sequence of only a single region of the bacterial genome, and some primers can exhibit a bias resulting in over- or under- representation of specific taxa (Rintala et al., 2017). By metagenomic shotgun sequencing, the entire genome of a specimen can be sequenced and analyzed, with more detailed information on the taxonomy and function of microorganisms being obtained.

In this study, 16S and metagenome sequencing were used to assess and compare salivary microbiome associated with SACC and healthy controls. In our results, metagenome shotgun sequencing allows the identification of a great larger number of taxa compared with 16S rRNA sequencing. Although alpha diversity analysis showed no difference in bacterial diversity and richness between the two groups, beta diversity analysis showed that the phylogenetic distance significantly separated SACC patients from healthy controls, indicating different bacterial community structures between the two groups.

It is acknowledged that potential unknown mechanisms relevant to carcinogenesis were associated with the microbial shifts, which may be provoked by the alterations in oral microenvironment that disturb the normal salivary microbiome (Wolf et al., 2017). Several bacteria were indicated to affect carcinogenesis in oral cancer. For example, *streptococci* can lower the local pH by generating short chain organic acids, and contribute to the production of an acidic and hypoxic tumor environment (Mazzio et al., 2010; Lunt and Vander Heiden, 2011). *Streptococcus* and *Neisseria* are reported to synthesize acetaldehyde, a well-known carcinogen (Moritani et al., 2015). Porphyromonas gingivalis and Fusobacterium nucleatum induce production of inflammatory cytokines, cell proliferation, and inhibition of apoptosis, cellular invasion, and migration thorough host cell genomic alterations (Chattopadhyay et al., 2019). A potential mechanism has been proposed in pancreatic cancer, indicating that lipopolysaccharide (LPS) of Gram-negative bacteria triggers an innate immune response that involves recognition by Toll-like receptor 4 (TLR4) and then activate the nuclear factor kB pathway, resulting in chronic inflammation and cancer development (Irfan et al., 2020).

In this study, the most prevalent taxa and taxa with significantly different relative abundances in SACC are consistent with several studies on oral squamous cell carcinoma (OSCC), suggesting that *Streptococcus* was the prevalent genus and increased significantly in oral cancer compared with *Prevotella* in the control group (Pushalkar et al., 2011; Pushalkar et al., 2012; Guerrero-Preston et al., 2016). In addition to *Streptococcus* and *Rothia*, LEfSE showed *Lautropia, Mycobacterium*, and *Propionibacterium* were also enriched in SACC while *Treponema, Bacteroides, Oribacterium*, and *Clostridium* were more abundant in healthy controls. It is suggested that bacterial products such as endotoxins, enzymes, and metabolic byproducts may alter the signaling pathway and induce host cell genomic alterations that drive proliferation. In addition to specific bacteria, their correlations might play a role in carcinogenesis, as SACC patients exhibited more complex and aggregated correlations among salivary microbiota than healthy controls, according to co-occurrence analysis. Further researches are needed to investigate the influences and mechanisms of the potential microbial biomarkers as well as the correlations among them.

KEGG is an encyclopedia of genes and genomes assigning functional meanings both at the molecular and higher levels (Kanehisa et al., 2017). The major categories of the KEGG pathway database are metabolism, genetic information processing, environmental information processing, cellular processes, organismal systems, human diseases, and drug development. In this study, functional annotation was performed based on KEGG database. The results showed vigorous metabolism in salivary microbiota, especially carbohydrate metabolism, which was consistent with previous studies on oral microbial functional prediction in oral disease and health. As shown in regression analysis, beta diversity of the functional profiles was significantly correlated with that of microbial community structures, providing evidence that overall functional attributes of communities may be altered by the shifts in microbial composition.

The LEfSE results indicated that SACC were associated with several KEGG pathways. The ATP-binding cassette (ABC) transporter is widely recognized to be related to multidrug resistance, and can influence the transport of xenobiotics, metabolites and signaling molecules across cell membranes. They are also suggested to have some impacts on the malignant potential of cancer cells *in vitro* and *in vivo* (Fletcher et al., 2016). Another membrane transport system, phosphotransferase system (PTS), is involved in the transport and metabolism of carbohydrates, and it is the most common type of carbohydrate-transport system in *Streptococcus mutans* (Ajdic and Chen, 2013). Two-component system (TCS) comprising sensor histidine kinases and response regulator proteins is important in bacterial and archaeal signal transduction, and plays vital roles in cellular survival, virulence, and cellular development (Zschiedrich et al., 2016). The detected biomarkers of biosynthesis of amino acids, glutathione metabolism, biosynthesis of valine & leucine & isoleucine, and 2-Oxocarboxylic acid metabolism indicated important changes in amino acid metabolism. Further studies are needed to investigate the specific mechanisms.

Microbes regulate the physiological and pathogenetic processes of human body by producing various CAZymes to degrade and modify complex carbohydrates and generate signal molecules for further utilization in human cells (Lombard et al., 2014). The observed CAZyme profile and microbial habitation have adapted to the local carbohydrate composition. Some GHs are biomarkers of dental plaque biofilm formation, and take part in polysaccharide synthesis (Cantarel et al., 2012). In our results, we figured out some biomarkers of SACC, including CBM50, GH1, CBM71, GT87, GH104 and GH13_31, and the potential impacts of them might be further researched.

In this study, Comprehensive Antibiotic Resistance Database (CARD) was used to provide data on the molecular basis of antimicrobial resistance. Among the six AROs enriched in SACC, bcrA and lmrD are described as efflux pump conferring antibiotic resistance, while murA, PBP2x, PBP2b and parC are antibiotic resistant gene variant or mutant. The global spread of antibiotic-resistant pathogens might increase the mortality of cancer patients. It is proposed that chemotherapy contributes to the emergence of antibiotic-resistant bacteria and drives pathogen overgrowth. Therapies which can restore the microbiota are needed to break the cycle of infection and treatment failure in cancer (Papanicolas et al., 2018).

The various combinations, organizations and expressions of virulence factors are responsible for the diverse clinical symptoms of pathogen infections. Cya is a kind of toxin of *B.pertussis* and T6SS is related to secretion system. Both of them are offensive virulence factors enriched in SACC, and further researches are needed to gain more indepth understanding.

In this study, we profiled the salivary microbiome of SACC patients and healthy controls by high throughput sequencing based on 16S rRNA as well as whole-genome shotgun metagenomics. Although the direct causality was not revealed, we found initial evidence that differences in microbial diversity, composition and function might inform disease status in SACC patients. The biomarkers detected need further researches to verify their effect in SACC and to search for early diagnostic and prognostic methods based on them. In addition, an enlarged group size will be needed to gain a more thorough understanding. However, despite these considerations, our results represent a significant first step in understanding the microbiological etiology of SACC, and lay a foundation for the development of new microbiome-connected diagnostic techniques.

## Data Availability Statement

The datasets presented in this study can be found in online repositories. The names of the repository/repositories and accession number(s) can be found in the article.

## Ethics Statement

The studies involving human participants were reviewed and approved by Ethics committee of Chongqing Medical University. The patients/participants provided their written informed consent to participate in this study. Written informed consent was obtained from the individual(s) for the publication of any potentially identifiable images or data included in this article.

## Author Contributions

QJ, XL, and QY contributed to research design, data acquisition, data analysis and interpretation, drafted and critically revised manuscript. LC contributed to data interpretation and critically revised the manuscript. DY contributed to conception and design, and critically revised the manuscript. All authors agreed to be accountable for all aspects of this work. All authors contributed to the article and approved the submitted version.

## Funding

This study was financially supported by the National Natural Science Foundation of China (31970783 to DY) and the Key Project of Yuzhong District Scientific and Technological Commission (20120201 to DY).

## Conflict of Interest

The authors declare that the research was conducted in the absence of any commercial or financial relationships that could be construed as a potential conflict of interest.

## Publisher’s Note

All claims expressed in this article are solely those of the authors and do not necessarily represent those of their affiliated organizations, or those of the publisher, the editors and the reviewers. Any product that may be evaluated in this article, or claim that may be made by its manufacturer, is not guaranteed or endorsed by the publisher.

## Supplementary Material

Supplementary Figure 1 The rarefaction curves. The x axis indicates the number of reads sampled. The y axis indicates the Shannon index of OTU level. Different colors represent different groups.
