# Supplementary figures and images for: Salivary Microbiome in Adenoid Cystic Carcinoma Detected by 16S rRNA Sequencing and Shotgun Metagenomics
Source: Front Cell Infect Microbiol. 2021 Dec 14;11:774453. doi: 10.3389/fcimb.2021.774453 (PMC8712576; doi:10.3389/fcimb.2021.774453)

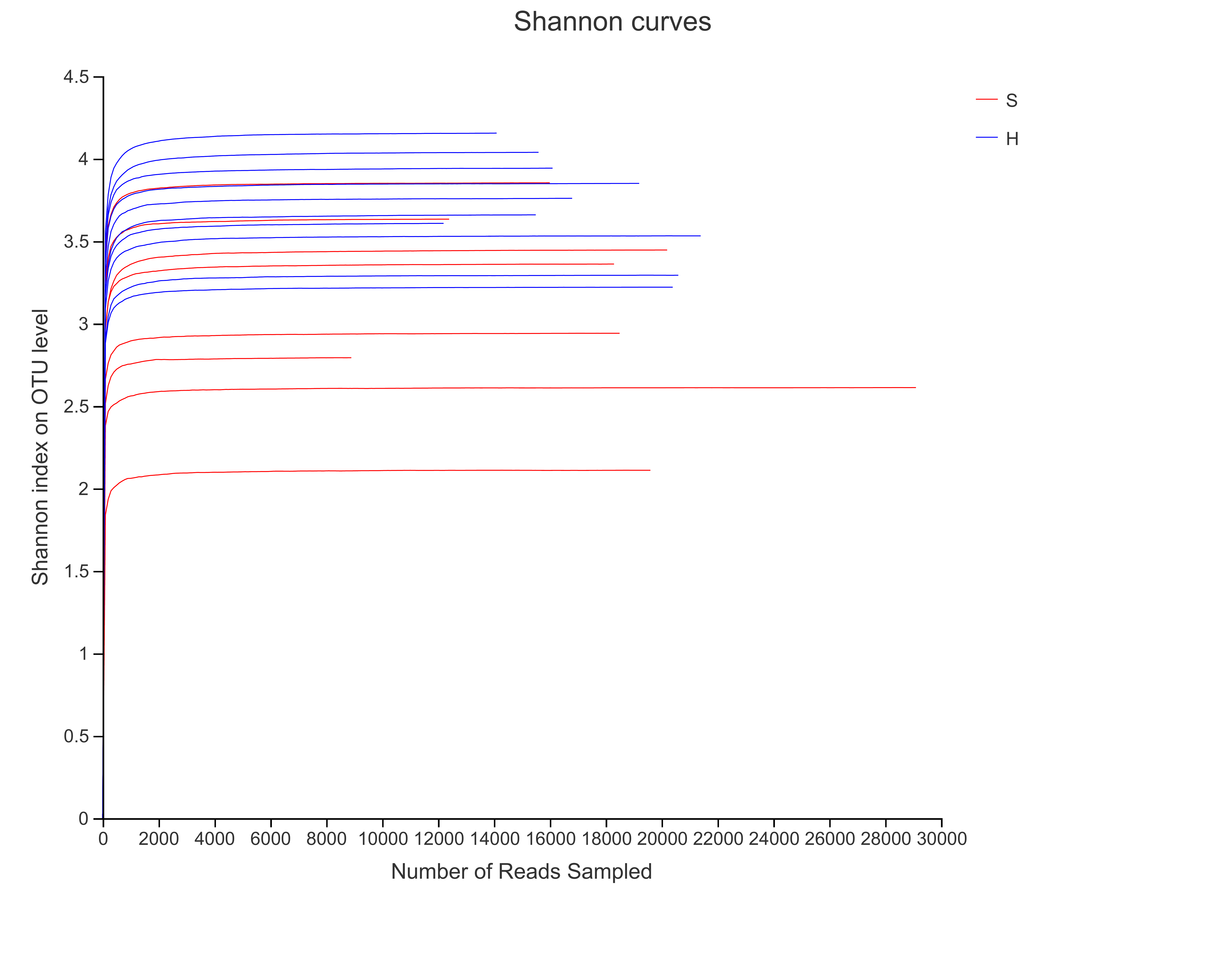

Supplement: Supplementary Figure 1 — The rarefaction curves. The x axis indicates the number of reads sampled. The y axis indicates the Shannon index of OTU level. Different colors represent different groups. [file Image_1.png]
